# Current recommendations for clinical surveillance and genetic testing in rhabdoid tumor predisposition: a report from the SIOPE Host Genome Working Group

**DOI:** 10.1007/s10689-021-00229-1

**Published:** 2021-02-03

**Authors:** M. C. Frühwald, K. Nemes, H. Boztug, M. C. A. Cornips, D. G. Evans, R. Farah, S. Glentis, M. Jorgensen, K. Katsibardi, S. Hirsch, K. Jahnukainen, I. Kventsel, K. Kerl, C. P. Kratz, K. W. Pajtler, U. Kordes, V. Ridola, E. Stutz, F. Bourdeaut

**Affiliations:** 1 Paediatric and Adolescent Medicine, Swabian Children’s Cancer Center, University Medical Center Augsburg, Stenglinstraße 2, 86156 Augsburg, Germany; 2 St. Anna Children’s Hospital and Children’s Cancer Research Institute, Department of Pediatrics, Medical University of Vienna, Vienna, Austria; 3 Department of Genetics, University Medical Center Utrecht, Utrecht, The Netherlands; 4 Manchester Centre for Genomic Medicine, Division of Evolution and Genomic Sciences, MAHSC, St Mary’s Hospital, Manchester University Hospitals NHS Foundation Trust, University of Manchester, Manchester, UK; 5 Department of Pediatrics, Division of Hematology/Oncology, LAU Medical Center-Rizk Hospital, Ashrafieh, Beirut, Lebanon; 6 Pediatric Hematology-Oncology Unit, First Department of Pediatrics, National and Kapodistrian University of Athens, “Aghia Sofia” Children’s Hospital, Athens, Greece; 7 Great Ormond Street Hospital for Children, NHS Foundation Trust, London, WC1N 3JH UK; 8 Institute of Human Genetics, Heidelberg University Hospital, Heidelberg, Germany; 9 Hopp Children’s Cancer Center Heidelberg (KiTZ), Heidelberg, Germany; 10 Children’s Hospital, University of Helsinki and Helsinki University Hospital, Helsinki, Finland; 11 Department of Pediatric Hematology-Oncology, The Edmond and Lily Safra Children’s Hospital, Chaim Sheba Medical Center, 52621 Tel-Hashomer, Israel; 12 Department of Pediatric Hematology and Oncology, University Children’s Hospital Münster, Albert-Schweitzer-Campus 1, 48149 Münster, Germany; 13 Pediatric Hematology and Oncology, Hannover Medical School, Hannover, Germany; 14 Division of Pediatric Neurooncology, German Cancer Research Center (DKFZ) and German Cancer Consortium (DKTK), Heidelberg, Germany; 15 Department of Pediatric Oncology, Hematology, and Immunology, University Hospital Heidelberg, Heidelberg, Germany; 16 Department of Pediatric Hematology and Oncology, University Medical Center Hamburg Eppendorf, Hamburg, Germany; 17 Department of Pediatric Oncology and Haematology, Mitera Children’s Hospital, Athens, Greece; 18 Department of Oncology, University Children’s Hospital, Zurich, Switzerland; 19 Institut Curie, SIREDO Pediatric Cancer Center, INSERM U830, Laboratory of Translational Research in Pediatric Oncology, Paris Sciences Lettres Research University, Paris, France

**Keywords:** Rhabdoid, ATRT, SMARCB1, Predisposition, Germline, Surveillance

## Abstract

The rhabdoid tumor (RT) predisposition syndromes 1 and 2 (RTPS1 and 2) are rare genetic conditions rendering young children vulnerable to an increased risk of RT, malignant neoplasms affecting the kidney, miscellaneous soft-part tissues, the liver and the central nervous system (Atypical Teratoid Rhabdoid Tumors, ATRT). Both, RTPS1&2 are due to pathogenic variants (PV) in genes encoding constituents of the BAF chromatin remodeling complex, i.e. *SMARCB1* (RTPS1) and *SMARCA4* (RTPS2). In contrast to other genetic disorders related to PVs in *SMARCB1* and *SMARCA4* such as Coffin-Siris Syndrome, RTPS1&2 are characterized by a predominance of truncating PVs, terminating transcription thus explaining a specific cancer risk. The penetrance of RTPS1 early in life is high and associated with a poor survival. However, few unaffected carriers may be encountered. Beyond RT, the tumor spectrum may be larger than initially suspected, and cancer surveillance offered to unaffected carriers (siblings or parents) and long-term survivors of RT is still a matter of discussion. RTPS2 exposes female carriers to an ill-defined risk of small cell carcinoma of the ovaries, hypercalcemic type (SCCOHT), which may appear in prepubertal females. RT surveillance protocols for these rare families have not been established. To address unresolved issues in the care of individuals with RTPS and to propose appropriate surveillance guidelines in childhood, the SIOPe Host Genome working group invited pediatric oncologists and geneticists to contribute to an expert meeting. The current manuscript summarizes conclusions of the panel discussion, including consented statements as well as non-evidence-based proposals for validation in the future.

## The genetics of Malignant Rhabdoid Tumors (MRT)

MRT are rare, highly aggressive embryonal malignancies affecting predominantly infants and rather young children often below 3 years of age. They may affect any anatomical structure, commonly the central nervous system (i.e. Atypical Teratoid Rhabdoid Tumor, ATRT) where > 50% arise in the cerebellum [1]. Further common anatomical sites include extracranial, extrarenal tissues (eMRT; e.g. head and neck, paravertebral muscles, liver, bladder, mediastinum, retroperitoneum, extremities, pelvis, and heart) and kidneys (RTK—rhabdoid tumor of the kidney).

MRT are characterized by a remarkably simple genome, with an extremely low number of single nucleotide variants per mega base and few recurrent PVs apart from those affecting the gene *SMARCB1*. *SMARCB1* encodes the protein BAF47, which is a core-member of the BAF chromatin remodeling complex. More than 90% of MRT harbor biallelic loss of function of *SMARCB1*; the few remaining cases show a loss of function of the *SMARCA4* gene, which encodes BRG1, the helicase/ATPase protein of the BAF complex [2]. Taken together, MRT are an aggressive malignancy of early childhood characterized by the disruption of the BAF complex in an otherwise stable genome.

Pedigrees with several affected siblings have for long suggested that this malignancy could occur in a cancer predisposition syndrome, Rhabdoid Tumor Predisposition Syndrome (RTPS) [3]. Ever since the first description and identification of PVs in *SMARCB1* and *SMARCA4* as causative genetic events, compelling evidence has accumulated that MRT are frequently associated with genetic lesions in the germline. Many aspects of the clinical care for patients with RTPS remain unresolved however, especially as little consistency may be deducted due to small case numbers.

The SIOP Europe Host Genome Working Group has held a consensus meeting to update our knowledge on RTPS and to address unresolved issues. In a group discussion among human geneticists, pediatric oncologists and biologists we first asked whether the lack of evidence should deter us from providing recommendations based on the current admittedly modest evidence. All participants supported communicating provisional guidelines, as the rarity of the disease and an urgent clinical need call for expert recommendations.

The current manuscript provides an overview of the current knowledge and summarizes main conclusions drawn from the panel discussion.

## RTPS1 and RTPS2: current knowledge

### Epidemiology

Among 384 patients registered to the European Rhabdoid Registry (EU-RHAB), the median age at diagnosis was 18 (0–211) months for ATRT (n = 244), 13.5 (0–207) months for eMRT (n = 89), 13 (2–166) months for RTK (n = 34) and 3 (0–23) months for synchronous multifocal MRT (n = 17) (M. Frühwald’s currently unpublished data). All available series report a male predominance with 1.3–1.5 male: 1 female. The age-standardized annual incidence rate is between five (extracranial rhabdoid tumors) and eight per million (ATRT) in children below 1 year of age and decreases to between 0.6 and 2.2 per million at 1 to 4 years [4, 5]. In the US, the annual incidence among children less than 15 years is 0.89 per million for ATRT, 0.32 per million for eMRT and 0.19 per million for RTK [6].

Rhabdoid Tumor Predisposition Syndromes (RTPS) are characterized by heterozygous germline PVs leading to inactivation of *SMARCB1* (commonly) or *SMARCA4* (rarely) which are inherited in an autosomal dominant fashion. Among newly diagnosed cases of MRT 25–35% carry a germline PV in *SMARCB1* (RTPS1) [7–9]. Given the rarity of cases with a *SMARCA4* germline PV the exact incidence of RTPS2 is unknown.

### Increased rhabdoid tumor risk in carriers of germline variants

In patients with RTPS, Rhabdoid Tumors have been reported in close to any anatomical localization [4, 5, 10]; in order of decreasing frequency: brain, kidney, soft tissues, liver, skin, and others.

**Fig. 1:**
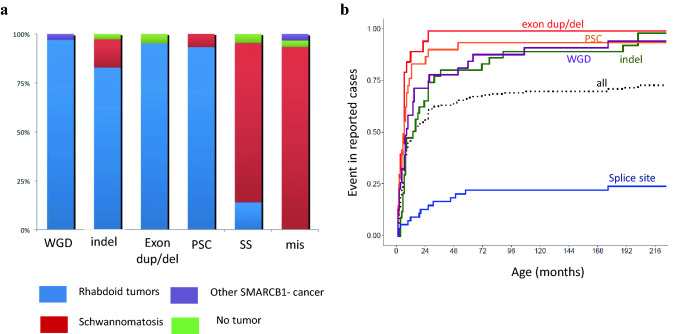
Epidemiological features of *SMARCB1* pathogenic variants (PVs), based on 179 cases reported in the literature. **a** Proportion of each phenotype observed with the various types of variants; *WGD* whole gene deletion, *indel* base insertion or deletion leading to frameshift, *ex.dup/del* exon duplication or deletion, *PSC* premature stop codon, *SS* splice site variant, *mis* missense variant. **b** Rhabdoid tumor free survival among patients screened for *SMARCB1* PV and reported in the literature; this illustrates the compilation of reported cases with a personal or family history of RT and a germline PV in *SMARCB1*. Penetrance might be biased toward that of high-risk individuals, but the compilation of reported cases with a personal or family history of RT and a germline PV in *SMARCB1*. *Sources* [3, 7, 8, 17, 20–22, 32–35, 45, 48, 57, 60, 73–82]
- Up to 1/3 of patients with RTPS have multiple (synchronous–multifocal) tumors, with bifocal manifestation most commonly in kidney and brain [8].

RTPS appear to be characterized by a few clinical features:

- Tumors may be detected pre- [11] or perinatally [12]; RTPS is diagnosed in about 66 to 80% of patients with congenital MRT [12, 13].
- Patients with RTPS are diagnosed at a median age of four to seven months (range: prenatally—60 months) compared to individuals with sporadic MRT (median age around 18 months, range: age 1 day—228 months) [14, 15]; almost all cases of MRT with germline mutations will be confirmed before the age of 2 years (Fig. 1b).

In patients with RTPS1 penetrance may be extremely high (e.g. > 90% by 5 years) [12, 14, 16]. This statement may however be based upon selection bias and larger series of systematically screened trios (parents and affected offspring) are needed to more precisely define penetrance.

We know even less about the penetrance of RTPS2. Wider use of gene panels including *SMARCB1* and *SMARCA4* among many other genes may reveal more carriers of a PV with no cancer phenotype. One needs to keep in mind the risk of ascertainment bias in these rare syndromes.

### Increased risk of other neoplasms in RTPS

Given the low survival rate and high penetrance in childhood the risk for subsequent cancers cannot yet be extrapolated with adequate accuracy. Only a few teenagers and adults with RTPS1 have been reported, either as long-term survivors or as, rather rarely, clinically unaffected carriers. Other tumor types including epithelioid schwannomas [17], malignant peripheral nerve sheath tumors [14], myeloid sarcoma [18], meningioma [19, 20], benign myoepithelioma [19], chondrosarcoma [21] and ganglioglioma [22] have been reported. Rarely, ATRT occur in adult mutation carriers, e.g. a sellar ATRT-like tumor was described in a 51 year-old mother of two children who died from MRT [23].

Intriguingly, RTPS1 and multiple schwannomatosis may overlap. *SMARCB1* germline PVs are responsible for about 40% of inherited “schwannomatosis” and 10% of apparently sporadic cases, characterized by the development of multiple indolent schwannomas [24]. Interestingly, even though these schwannomas show some rhabdoid features and loss of INI1/BAF47 staining, they are distinct from MPNST with SMARCB1 loss. Rare patients/pedigrees affected by both tumor types have been observed, in line with a rather robust genotype–phenotype correlation [17, 25, 26]. While missense PVs and exon 1 frameshifts that cause a re-initiation codon are associated with schwannomatosis, large deletions and premature stop codons resulting from nonsense PVs and intragenic deletions almost exclusively predispose to MRT (Fig. 1a). A few contradictory examples have been reported. Notably, hypomorphic variants may occasionally induce both MRT and schwannomatosis, synchronously or metachronously [17, 25] PVs affecting splice sites have been encountered in both conditions.

Given its rarity, the cancer spectrum in RTPS2 has not been fully defined yet. However, RTPS2 and Small Cell Carcinoma of the Ovary, Hypercalcemic type (SCCOHT) predisposition syndrome share the same genetic germline abnormalities and a link between these two entities was established. Some authors have proposed to rename SCCOHT as “Rhabdoid tumors of the ovary”. In order to accommodate for the rare SMARCB1-negative ovarian rhabdoid tumors we have chosen to keep the term as it is until further defining analyses have been presented.

Within two independent families affected by MRT as well as SCCOHT, a *SMARCA4* germline PV was demonstrated [27, 28]. One might speculate that female survivors of RTPS2 in infancy could later develop SCCOHT, which has been demonstrated in prepubertal females [29]. It is noteworthy that no patient with SCCOHT published to date developed MRT during infancy. Apart from SCCOHT, germline truncating PVs of *SMARCA4* have also been associated with undifferentiated uterine sarcomas [30] and a single case of BRG1/SMARCA4-deficient lung carcinoma [31].

### Specific phenotype of distal congenital 22q11.2 deletion

In some cases, RTPS1 may be due to a distal deletion of 22q11.2, encompassing *SMARCB1*. The phenotype is variable depending on the extent of the deletion, but may consist of intra-uterine and post-natal growth retardation, speech delay, behavioral problems, and minor dysmorphic features [22, 32, 33]. The largest genomic deletions are associated with a phenotype overlapping with the velo-cardio-facial syndrome [33–35]. A definitive cancer risk cannot be extrapolated for patients with distal 22q11.2 deletion syndrome, but deletions encompassing *SMARCB1* should receive vigilant surveillance from birth to adulthood as large deletions may predispose to late occurrence of MRT [22, 36].

### Coffin Siris syndromes and an associated risk for neoplasm

**Fig. 2:**
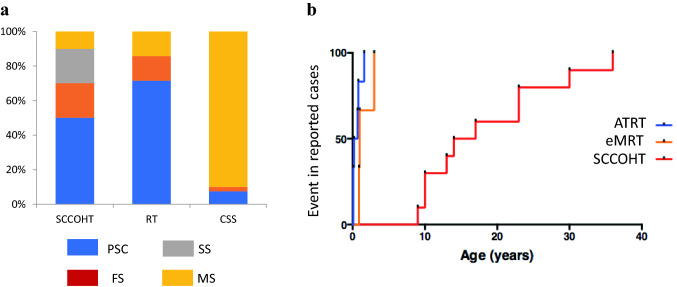
Epidemiological features of *SMARCA4* pathogenic variants, based on 70 cases (65 published, 5 author’s own unpublished cases). **a** proportion of each phenotype observed with the various types of variants; **b** rhabdoid tumor (orange line: eMRT; blue line: ATRT) and SCCOHT (red line) free survival among patients screened for *SMARCA1* PV and reported in the literature; this graph does not illustrate the real penetrance, but the compilation of reported and personal cases with a personal or family history of RT and a germline PV in *SMARCA4. Sources*: [9, 27, 41, 83–86]

Coffin Siris syndrome (CSS) is a rare genetic disorder characterized by learning difficulties, coarse facial features, hypertrichosis, and hypoplasia of the fifth digits/nails of the hands and feet. A minority of cases is due to a PV in *SMARCB1* or *SMARCA4*. Most PV in *SMARCB1* are in-frame deletions within exon 9, a genotype exclusively reported in CSS and thus far not related to any malignancy. Another substantial number of cases of CSS are due to missense PV in the last 2 exons of *SMARCB1* [37], quite similar to those reported in schwannomatosis. Consistently, a patient with CSS affected by schwannomatosis and a missense PV in *SMARCB1* has been reported [38]. However, patients with MRT are missing from the literature indicating a specific tumor risk associated with this rare genotype in CSS. A few additional cases of CSS have been linked to *SMARCA4* PV. In a series of 15 patients with CSS  and *SMARCA4* PV, Li et al. reported two individuals with nonsense variants, associated with a milder phenotype; in the two male patients no tumor was reported by ages 9 and 15 years respectively [39]. By contrast, one patient with a mild CSS phenotype related to a truncating variant c.2935C > T;p.Arg979* developed a SCCOHT [40], again suggesting that detection of truncating SMARCA4 variants in females justifies surveillance for SCCOHT [40], suggesting that detection of this particular genotype in females justifies surveillance for SCCOHT. None of the 13 patients with a missense *SMARCA4* PV in the series by Li et al. developed any tumor; similarly, no tumor was detected in the series by Sekiguchi et al. reporting 7 patients (5 males) with all missense variants in *SMARCA4* [37]. Reciprocally, at least two SCCOHT patients without other signs of CSS harbor ‘CSS-like’ missense PV in the helicase domain of *SMARCA4* [41] (Fig. 2a). Altogether, the risk in CSS to develop tumors seems to be low, but remains difficult to estimate [42, 43]. Specific genotypes might need surveillance.

### Outcome of patients with RTPS1 and RTPS2

Prognosis of MRT in the frame of RTPS appears to be inferior to sporadic tumors [16, 44], and this seems to be true for *SMARCB1* as well as for *SMARCA4* associated RTPS. While rhabdoid tumors associated with RTPS1 exhibit an overall survival of 10–20% [7, 44], a total of six out of seven patients with ATRT and RTPS2 died within 20 months in the series by Hasselblatt et al. [9] (Fig. 2b). The dismal prognosis may be partly related to the early onset of MRT, and a frequent multifocal presentation.

## Screening for germline PV in *SMARCA4* and *SMARCB1*

### Why should all patients affected by MRT be tested?

Participants of the consensus meeting agreed on the utility of genetic counseling and testing in patients with MRT.

The main purpose of genetic testing will be

1. to answer questions regarding the potential genetic cause of the disease,
2. to reassure parents about the absent (or low) MRT risk in siblings, when no PV can be identified in the germline,
3. to offer prenatal diagnosis in cases of RTPS in the proband, and finally
4. to offer a cancer surveillance schedule.

Reports on late occurrences of secondary rhabdoid and other tumors in patients cured from a first MRT have been accumulating [45–47]. This further justifies systematic genetic screening for all patients with MRT. It also illustrates that SMARCB1-deficient non-MRT neoplasms may be part of the RTPS spectrum.

### Should we test patients with SMARCB1-deficient non rhabdoid tumors?

SMARCB1-deficient non-MRT neoplasms of childhood represent an expanding spectrum of diseases including highly aggressive but also low-grade neoplasias [19, 48] such as undifferentiated chordomas, epithelioid sarcomas, epithelioid MPNST, and other rare entities [49]. One case of a *SMARCB1* PV with SCCOHT is also on record [50]. Employing readily available BAF47 immunostaining, SMARCB1-deficient tumors may be rapidly identified. At present, there is no evidence in the literature that SMARCB1-deficient cancers other than MRT necessitate genetic counseling and testing. In particular, no familial cases of epithelioid sarcomas or undifferentiated chordomas have been reported so far. Nonetheless, the panel argues that patients with “SMARCB1-deficient non-RT neoplasms” should be offered genetic testing on a research basis and, if positive for a PV, cancer surveillance ideally in the setting of a tumor predisposition clinic. Regardless of the fact that little is known about the penetrance of non-rhabdoid SMARCB1-deficient tumors in RTPS, it has been generally accepted that any rare cancer in childhood justifies discussion and referral to genetic testing. More evidence from accumulated data will help to establish the clinical and thus individual benefit in the future [51–54].

### Should we test unaffected relatives?

Agreement has been reached that clinical surveillance should include pre-symptomatic carriers especially when a first-degree relative has been diagnosed with active disease. This initially includes the affected patients’ parents. Identification of a PV in an unaffected parent remains an exceptional event. Nevertheless, familial recurrence despite unremarkable sequencing results in the parents has occasionally been reported, suggesting gonadal mosaicism [3, 7, 8, 55]. Thus, siblings of children with RTPS should be offered genetic screening as well, provided that clinical surveillance will be accepted, in case of detection of a PV in the germline. Given the rather high penetrance, the young median age at tumor occurrence and the severity of the disease, it is justified to discuss prenatal diagnosis in parents and siblings once RTPS has been identified in the proband [55].

### Who should be tested for RTPS2?

Regarding *SMARCA4*, there is an agreement to recommend systematic genetic counseling to all children with either BRG1-deficient MRT or SCCOHT. This statement is justified due to the serious impact of knowing about a *SMARCA4* PV for all females in a pedigree. Recommendations for patients with SCCOHT and families without MRT have been developed and are discussed elsewhere [56].

## Molecular testing and interpretation of the risk according to PV type

### Molecular testing: what should not be missed?

*SMARCB1* comprises nine exons and produces a 1.749-bp transcript variant 1 which encodes the isoform A (RefSeq NM_003073.5). Nonsense, frameshift, whole and partial gene deletions have been reported in RTPS1. Hence, genetic screening should allow for the identification of any of those PV types. Classical approaches combine Sanger sequencing of the nine coding exons and the intron-to-exon boundaries plus multiplex ligation-dependent probe amplification (MLPA) to identify intragenic deletions or duplications. Such approaches may be replaced by high-coverage dedicated next-generation sequencing techniques spanning all exons of *SMARCB1* supplemented by copy number analyses.

Sequencing coding sequences may only miss deep intronic PVs in the germline. At least one deep intronic hot-spot has previously been reported in intron 1 [57]. In consequence, we recommend that intron 1 should be incorporated in future NGS screening approaches. In general, identification of the two inactivating genetic events in a tumor genome is recommended before any conclusion may be drawn on the germline status. Moreover, the augmented depth of sequencing may support the discovery of a low frequency of mosaicism, which otherwise will escape the detection threshold of Sanger sequencing (< 10%).

For *SMARCA4*, multiple transcript variants encoding different isoforms have been described. By convention, PVs are numbered based on the sequence of the transcript encoding the longest isoform, comprising 36 exons (RefSeq NM_001128849.1). Given the length of the gene, capture-based sequencing facilitates genetic screening and will in most circumstances now be preferred over Sanger techniques.

### The interpretation of PV: what is their impact on cancer surveillance?

The interpretation of the variants will follow classical algorithms [58, 59]. However, it should be noticed that RTPS1 is almost exclusively associated with highly deleterious variants, i.e. those inducing inactivating truncation or copy loss [60].

Thus, the need for surveillance is undisputed in cases of premature stop codons, insertions and deletions leading to frameshift or whole gene loss. This is not at all clear-cut in the case of variants affecting splice sites as most of these variation types have been associated with schwannomatosis only (Fig. 1a). Nonetheless, compiled data from the literature estimate the risk of MRT with splice-site PV above 10%, encouraging similar surveillance for affected patients even if the penetrance appears less pronounced (Fig. 1b). The impact of the 3′ or 5′ location of such PV and the hypothetical level of residual normal protein has not been assessed, but may affect the tumor phenotype [61, 62]. On the contrary, missense variations have been associated with schwannomatosis and surveillance should follow published recommendations [63]. Thus far only one case of a missense PV (c.1142C>G; p.Thr381Arg) has been described in a patient suffering from a CRINET, cribriform neuroeptithelial tumour [16]. Due to the rarity of this event, the incidental finding of a missense PV, which does not cause inactivation of the protein in a tumor-free patient will likely not lead to an increased risk of malignancy and may not prompt the intense surveillance recommended for patients with RTPS. However, in case of the unsolicited finding of a *SMARCB1* missense variant predicted to be pathogenic, we strongly recommend the involvement of experts to determine the clinical relevance of the variant.

## Surveillance strategies for patients with RTPS1 and RTPS2

The paucity of reliable clinical data poses a major challenge in defining genetic screening and clinical surveillance recommendations for unaffected PV carriers (i.e. detected incidentally or by targeted analyses of families), and for patients who have already gone through a tumor disease and who survived.

### Review of current screening recommendations for RTPS1

As knowledge on RTPS has emerged and technology advanced (e.g. whole-body MRI, NGS technology) detailed surveillance guidelines for RTPS have been provided among others by Teplick et al., Foulkes et al. and Nemes and colleagues [64–66].

While Teplick et al*.* and Nemes et al*.* dichotomized their recommendation to children below 1 year and those between 1–4 (5) years, Foulkes and colleagues focused on all patients below 5 years of age. The recommendations likely reflect different health care systems as well as different institutional preferences. Teplick and Nemes recommend monthly head ultrasound in patients below 1 year, Foulkes on the other side proposes MRI of the CNS in three-monthly intervals. In contrast, while Nemes et al*.* suggest whole-body MRI every 3 months in patients at risk between 1 and 5 years of age, Foulkes and colleagues leave the issue open.

Very recently an excellent status paper on the current evidence, practical clinical approach for genetic testing and surveillance as well as research issues in SCCOHT has been published. We would like to point the reader to this manuscript for into-depth information and advice [56].

### What screening tools should be used?

Given the unpredictable location of MRT anywhere in the body, whole-body MRI (WBMRI) is an option, provided that it can be complemented with a CNS MRI for more accurate assessment of the brain and the spine [67]. The use of WBMRI, including legs and arms, clearly depends on the availability and resources of the respective national health care system and institution; where WBMRI is not available, clinical examination supplemented by ultrasonography and CNS MRI can be discussed as an alternative.

### Screening schedule for RTPS1—a proposal of the SIOPE Host Genome Working Group

Different rules may apply for MRT survivors, children being treated for an MRT, and unaffected carriers. For unaffected carriers, surveillance should start as soon as possible, i.e. at birth for newborns diagnosed through prenatal diagnosis or with 22q11.2 distal deletion syndrome. Considering the rapidity of MRT development for infants and other very young children, the SIOPE host genome working group suggests choosing surveillance intervals at close time points. The main objective is to diagnose tumors at a stage on which a complete resection is still possible (Tables 1 and 2).

**Table 1:** Current general recommendations for clinical surveillance in RTPS1 and RTPS 2

(1) Evaluate all patients with a signal tumor (i.e. Rhabdoid Tumor or SCCOHT) for a germline (GLM) mutation regardless of age	
(2) Offer expert genetic counselling to all patients with a PV in the GLM in *SMARCB1* or *SMARCA4* regardless of age	
(3) Offer genetic testing and counselling to all first-degree relatives of a patient with a PV in the GLM in *SMARCB1* or *SMARCA4* regardless of age; this may include prenatal testing	
(4) Initiate clinical surveillance of patients and first-degree relatives with a PV in *SMARCB1* or *SMARCA4* as early as possible (e.g. at birth)	
(5) Clinical surveillance should at least consist of ultrasound and clinical examination (i.e. neurological and developmental exam). Intervals for whole-body and CNS MRI (and the subsequent need for anesthesia or deep sedation) vary widely. They depend on institutional and/or health care system resources but also on physician/proband preference

**Table 2:** Proposal for surveillance program for patients/probands with RTPS 1 (i.e. with a *SMARCB1* PV in the GLM)

Age	Type of exam	Intervals (comment)^b^
All	Whole body MRI (WBMRI)	At diagnosis for all patients with PV^c^ in *SMARCB1*
0–6 mos	MRI CNS incl. spine or CNS ultrasound or WBMRI^a^	Every 4 weeks, not less than every 2–3 months^d^
	Ultrasound abdomen plus soft tissues (e.g. neck)	
	Thorough clinical exam incl. neurologic	
7–18 mos	MRI CNS incl. spine	Every 2–3 months
	Ultrasound abdomen plus soft tissues (e.g. neck)	
	Thorough clinical exam incl. neurologic	
19 mos–5 years	MRI CNS incl. spine	Every 3 months
	Ultrasound abdomen plus soft tissues (e.g. neck)	
	Thorough clinical exam incl. neurologic	
> 5 years	Whole body MRI	Yearly
	Physical examination	Every 6 months

^a^Depending on resources and need for anesthesia

^b^Recommendations are subject to continuous adaptations

^c^Patients with a missense mutation may need distinct surveillance

^d^This intensive surveillance program may not be possible in many instances and potentially has to be made part of research projects; the panel justifies the proposal by the fact, that the risk for tumor occurrence is highest within this age group and timeframe

Up to 6 months of age we would mandate thorough clinical examination, including subcutaneous tissues at intervals of 4–6 weeks in a specialized facility. As MRI may be possible without general anesthesia in many instances, we suggest to regularly image by WBMRI and MRI of the CNS. The panel argued in favour of a 4–6 weeks interval wherever possible, not exceeding 2–3 months in any case. As such an intense surveillance program will be needed only on rather rare occasions but will likely still not be possible in many health care systems, we suggest to make an attempt to make it part of a research program wherever feasible.

In older children, the need for anesthesia or sedation becomes an issue and the refering physician may decide against imaging modalities such as WBMRI and CNS MRI at close intervals. Alternatively, instead of WBMRI, CNS MRI could be coupled with abdominal ultrasonography and careful clinical examination of all soft-tissue parts. Keeping a close 4–6-week interval for imaging beyond 6 months of age appeared unrealistic to the panel, who proposed to increase the intervals to 2–3 months, lasting until at least 36 months of age (Table 2).

The risk of developing a new onset RT dramatically decreases after 5 years of age [8]. It remains worthwhile, however, to screen individuals with RTPS, for MRT as well as for other manifestations (e.g., schwannomas, meningiomas, MPNST) [68, 69]. A practical approach includes physical examinations every 6 to 12 months with targeted imaging for symptomatic areas (e.g., neurologic deficit, change in physical features, menstrual disturbances), ideally in the setting of a tumor predisposition clinic [70]. CNS MRI and ultrasonography may be discussed.

In unaffected carrier parents, the risk for MRT is completely unevaluated. Nonetheless, a few case reports exemplify the remaining threat throughout life [7, 21, 23, 36]. Whether this justifies long-term systematic clinical surveillance could be debated given the presumably rather low risk. However, one can assume that education for early medical advice in case of symptoms, and early targeted imaging, is a minimalistic option.

### Discussion regarding surveillance for MRT in patients with RTPS 2

In a meta-analysis of publicly available data sets, Holsten and colleagues detected *SMARCA4* PVs as the cause of a rhabdoid tumor in 8/60 PV carriers, indicating incomplete penetrance [16]. Hence, patients with a *SMARCA4* germline PV seem to develop MRT with a much lower incidence than patients with a *SMARCB1* germline PV. However, the prognosis of children affected by RTPS2 seems to be as poor or even poorer than the one for patients with RTPS1 [9]. This suggests offering similar clinical surveillance to these patients as the one described for RTPS1 (Tables 1 and 2).

Consistently, germline investigation and if applicable surveillance should be proposed to all first-degree relatives of patients affected by SCCOHT who are below 36 months of age. International sharing of clinical experience is deeply warranted to evaluate whether such a proposal is realistic and actually brings any benefit to patients and their families.

As SCCOHT affect females from 5 to 46 years of age, clinical surveillance for RTPS2 individuals may not stop after 5 years of age, but rather change focus from the RT spectrum to include the ovaries. In children and teenagers, abdominal-pelvic ultrasonography will be preferred since it most often remains sufficiently informative in this age range. The issue of prophylactic risk reducing bilateral salpingo-oophorectomy (RRBSO) and its medical as well as ethical ramifications deserve major attention and interdisciplinary approach not only towards counselling but also towards research [71, 72].

## Conclusion: research issues

While the necessity of a clear guidance for clinicians is undoubted, it has to be kept in mind that the current guidelines rely on limited data and will remain “work-in-progress” until sufficient real-world evidence can be put forward.

Unresolved issues that deserve further international research endeavors include:

1. To demonstrate the clinical benefit of the surveillance guidelines for unaffected carriers; given the rarity of such conditions, international data sharing is warranted.
2. To follow up on aspects of feasibility, psychological burden and cost of such recommendations.
3. To evaluate the penetrance in children with 22q11.2 deletions syndromes encompassing *SMARCB1*; working with geneticists who follow these patients for non-tumor symptoms is critical.
4. To evaluate the incidence of mosaicism in the MRT population, and in parents of probands with MRT; to evaluate the tumor risk in case of mosaicism.
5. To evaluate the risk of MRT in offspring of patients with SCCOHT and vice versa; to search for putative modifiers that may influence the risk for one or another tumor type; to support decisions about the risk reducing prophylactic salpingo-oophorectomy [56].
6. To evaluate the tumor spectrum and tumor risk in older children, teenagers and adult pre-symptomatic carriers; to specify how long surveillance is needed.
7. To determine the actual need for germline testing in children with non rhabdoid SMARCB1-deficient tumors.

The SIOPe HGWG and other SIOP collaborative groups will now strive to set up an international network allowing to address these issues and to further evaluate how to better help families affected by such a devastating malignancy.
